# Clinical study status of diabetic gastrointestinal diseases

**DOI:** 10.3389/fendo.2025.1568552

**Published:** 2025-04-09

**Authors:** Xuechun Fan, Yanyan Wang, Jingsi Cao, Jing Yu, Jiaxing Tian, Jia Mi

**Affiliations:** ^1^ College of Traditional Chinese Medicine, Changchun University of Chinese Medicine, Changchun, Jilin, China; ^2^ Department of Endocrinology, The Affiliated Hospital to Changchun University of Chinese Medicine, Changchun, Jilin, China; ^3^ Institute of Metabolic Diseases, Guang’anmen Hospital, China Academy of Chinese Medical Sciences, Beijing, China

**Keywords:** diabetic gastrointestinal diseases, diabetic gastroparesis, clinical study, study status, clinical design

## Abstract

**Background:**

Diabetic gastrointestinal diseases not only affect the quality of life of patients, but also bring heavy economic burden to patients. Understanding of the current features of diabetic gastrointestinal diseases-related clinical trials are important for improving designs of clinical trials and identifying neglected areas of study. Despite the high prevalence of gastrointestinal complications among diabetic patients, comprehensive analyses of registered clinical trials are lacking. This study aimed to present a scoping overview of diabetic gastrointestinal diseases-related clinical trials registered in ClinicalTrials.gov, ChiCTR and high-quality diabetic gastrointestinal diseases-related clinical trials published in Pubmed in the past 10 years.

**Methods:**

The trials registered in ClinicalTrials.gov and ChiCTR databases from the establishment of the database to June 14,2024 were searched. Moreover, high-quality trials with impact factors of 5 points or more published in Pubmed from June 2014 to June 2024 were searched. The results were extracted and presented in tabular form.

**Results:**

Most studies focused on diabetic gastroparesis, with drug interventions being the most common. In addition, most studies were small sample sizes (≤100), randomized parallel controlled trials and 69.01% of the studies used different methods of blinding. Most studies did not conduct safety evaluation and follow-up.

**Conclusion:**

The diagnostic criteria of diabetic gastrointestinal diseases were diverse. Furthermore, most studies on diabetic gastrointestinal diseases focused on diabetic gastroparesis. There was considerable heterogeneity in study designs and efficacy evaluations.

## 1 Introduction

Diabetes is a chronic disease. According to the International Diabetes Federation (IDF) Diabetes Atlas, the global prevalence of adult diabetes was 10.5%, rising to 12.2% in 2045 (1). Gastrointestinal diseases are common in patients with diabetes mellitus (2). Up to 30% -70% of diabetic patients present intestinal-related dysfunction and complications (3). In addition to diabetic gastroparesis, diabetic gastrointestinal diseases also includes diabetic dyspepsia (4). Patients with diabetic gastrointestinal diseases will experience a number of gastrointestinal symptoms. Common gastrointestinal symptoms include nausea, vomiting, early satiety, abdominal bloating, diarrhoea, etc (5). Gastrointestinal symptoms affect quality of life in diabetes negatively (6). In addition, gastrointestinal symptoms also increase diabetes-related health care costs and bring a heavy economic burden to diabetic patients (7). More and more clinical workers had carried out clinical research on diabetic gastrointestinal diseases. Previous trials have mostly focused on gastroparesis and prokinetic therapies, but broader gastrointestinal symptoms manifestations remain underexplored. Moreover, there were great differences in research designs, such as the lack of standardized diagnostic criteria; limited follow-up periods and sparse data on interventions beyond prokinetics and so on.

Clinical trials are the basis of evidence-based medicine and the driving force of medical development (8). Clinical trial registration is an effective measure to promote the transparency of clinical trial design and implementation (9). ClinicalTrials.gov was a web-based registry maintained by the National Library of Medicine and National Institutes of Health (8). Meanwhile, the ClinicalTrials.gov database provided the most comprehensive information about ongoing and completed clinical studies worldwide (10). The Chinese Clinical Trial Register (ChiCTR) was sponsored and established by the Ministry of Health of China in June 2007, which was accepted as one of the main registrars of the World Health Organization International Clinical Trial Registry Platform (WHO ICTRP) (11). PubMed, the most widely used biomedical literature search engine, currently contains over 36 million articles (12). Although there are related reviews on diabetic gastroparesis, enteric neuropathy in diabetes and other diseases, there is lack of comprehensive reviews on clinical trial designs and outcomes for diabetic gastrointestinal diseases (13, 14). Physicians still lack a comprehensive understanding of high-quality clinical trials on diabetic gastrointestinal diseases.

A better understanding of the current research situation of clinical trials related to diabetic gastrointestinal diseases is very important for improving the design of clinical trials and the quality of research. Therefore, the purpose of this scoping overview is to examine how diabetic gastrointestinal disease trials are currently designed, executed, and reported, and to identify recommendations for future research.

## 2 Methods

### 2.1 Search strategy

The study used words related to search term “diabetes gastroparesis” or “diabetic gastroparesis” or “diabetic gastropathy” or “diabetes mellitus gastroparesis” or “diabetic gastrointestinal disorders” or “gastrointestinal diseases of diabetes” or “gastrointestinal disorders in diabetes” or “diabetic stomach” or “diabetic enteropathy” to retrieve the trials registered in ClinicalTrials.gov and ChiCTR databases from the establishment of the database to June 14,2024. High-quality trials with impact factors of 5 points or more published in Pubmed from June 2014 to June 2024 were searched with the same search terms. At the same time, clinical trials and randomized controlled trials were selected on the pubmed article type filter to initially include clinical studies related to diabetic gastrointestinal diseases.

### 2.2 Inclusion and exclusion criteria

The study included relevant clinical trials on diabetic gastrointestinal diseases if these studies 1) were conducted on humans, 2) were retrieved on ClinicalTrials.gov and ChiCTR databases from inception to June 14, 2024, 3) were high-quality clinical studies published in Pubmed from June 2014 to June 2024 (impact factor 5 and above).We did not include studies published on pubmed 10 years ago, because the study is to let clinicians understand the current research situation of diabetic gastrointestinal diseases. Moreover, the older articles are of little significance to understand the recent research status and are inconsistent with the purpose of this study. The study excluded surgical studies and studies unrelated to diabetic gastrointestinal diseases.

### 2.3 Data extraction

The retrieved clinical trials were strictly screened according to the inclusion and exclusion criteria. In addition, the following variables that coincidental studies were extracted using the Microsoft Excel table: sample size, diagnostic criteria, intervention type, follow-up duration, study design, and outcome measures. The items that could not be classified in the variables were listed as “other”. Data extraction was performed independently by two reviewers, with discrepancies resolved by consensus.

### 2.4 Statistical analysis

**Figure 1:**
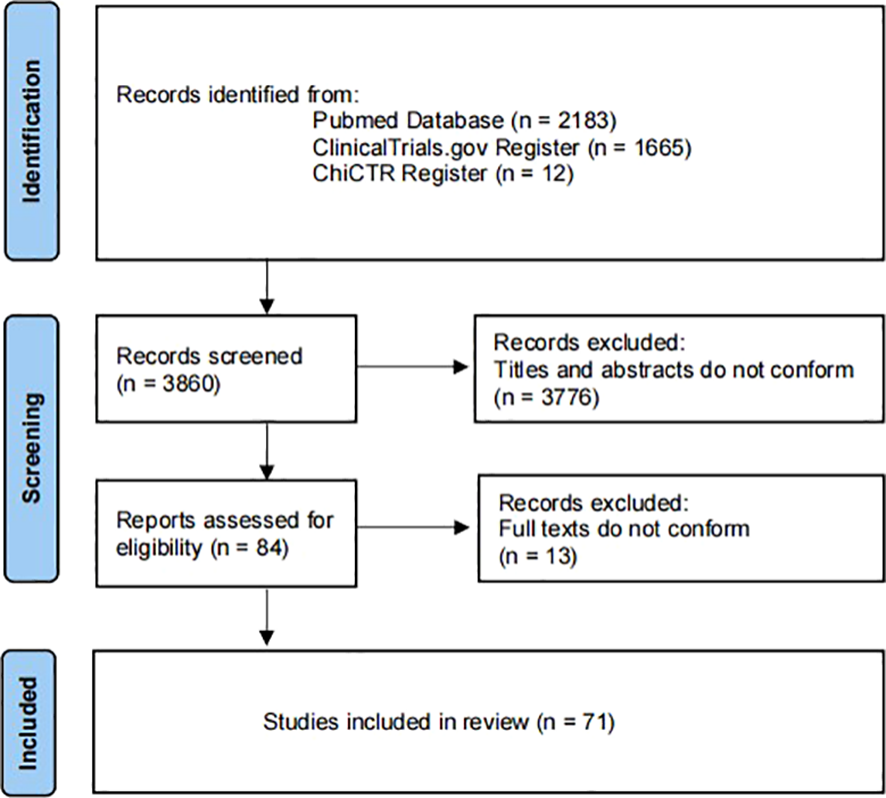
PRISMA flowchart of search.

**Figure 2:**
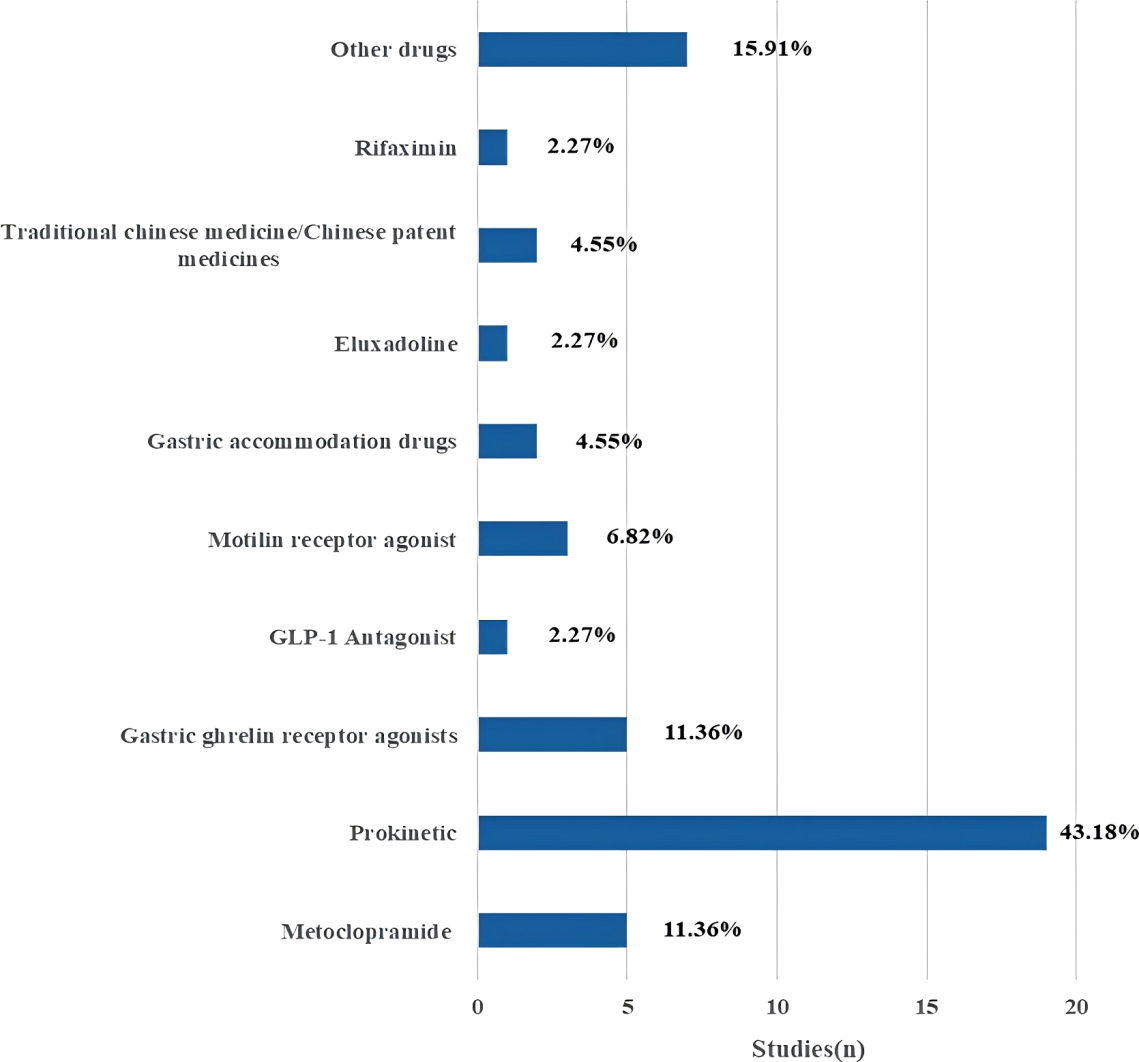
Type of Drug interventions.

Excel software was used for statistical analysis. This study is a scoping overview of diabetic gastrointestinal diseases-related clinical studies. Therefore, descriptive statistics were performed on the study contents extracted from the included articles in the form of composition ratio and frequency. Furthermore, “**Figure 1**” for study flow, “**Figure 2**” for types of drug intervention and “**Tables 1**-**4**” for detailed data. **Figure 1** flowchart follows the PRISMA guidelines (15).

**Table 1:** Inclusion criteria for studies on diabetic gastrointestinal diseases.

Characteristics	Category	N=71	Percentage of Total Records (%)
Diagnostic criteria	Score	30	42.25
	Delayed gastric emptying	41	57.75
	According to the diagnostic standards	13	18.31
	History of past illness	45	63.38
	Diagnosed as diabetes	61	85.92
	Symptom	14	19.72
	Exclude obstruction	21	29.58
	Others	5	7.04
	N/A	1	1.41
Intervened group	Mild to severe diabetic gastroparesis	11	15.49
	Mild to moderate diabetic gastroparesis	3	4.23
	Moderate to severe diabetic gastroparesis	14	19.72
	Moderate or severe diabetic gastroparesis	2	2.82
	Type 1 diabetic gastroparesis	3	4.23
	Diabetic gastrointestinal diseases	7	9.86
	Diabetic gastroparesis	31	43.66
Target size	0 to 30	24	33.80
	31 to 50	11	15.49
	51 to 100	12	16.90
	101 to 300	15	21.13
	301 to 500	7	9.86
	N/A	2	2.82
Age	18 to 60 years old (adult)	4	5.63
	18 years old and above (adult, elderly)	66	92.96
	N/A	1	1.41
Gender	All	66	92.96
	Male	1	1.41
	Female	4	5.63

N/A indicates no.

**Table 2:** Intervention characteristics for studies on diabetic gastrointestinal diseases.

Characteristics	Category	N=71	Percentage of Records (%)
Intervention time of trials	Within 24 hours	9	12.68
	One week and within one week	5	7.04
	Four weeks and within four weeks	24	33.80
	Eight weeks and within eight weeks	10	14.08
	Twelve weeks and above	18	25.35
	Not always	2	2.82
	N/A	3	4.23
Follow-up time	14 days and less than 14 days	4	5.63
	4 weeks and above	4	5.63
	14 days (+/-2 days)	1	1.41
	N/A	62	87.32
Intervention/Treatment	Drug	44	61.97
	Iiquid Nutrient	2	2.82
	Fecal Microbiota Transplantation	3	4.23
	Acupuncture	6	8.45
	Inspection/Equipment	11	15.49
	Diet	1	1.41
	Other	3	4.23
	NO	1	1.41

N/A indicates no.

## 3 Results

### 3.1 Study selection

A total of 71 clinical trials were included in the study (**Figure 1**). The initial search yielded 3860 records from ClinicalTrials.gov, ChiCTR and Pubmed. Of these, 3776 were excluded as they were repetitive and did not meet the defined inclusion criteria. Next, the full texts of the remaining 84 trials were screened. Of these, 13 were excluded as they were not diabetes-related gastrointestinal diseases. Finally, leaving 71 clinical trials for inclusion.

### 3.2 Inclusion criteria for studies on diabetic gastrointestinal diseases

Inclusion criteria for studies on diabetic gastrointestinal diseases were listed in **Table 1**. In the diagnostic criteria of diabetic gastrointestinal diseases, 61 studies (85.92%) clearly mentioned the diagnosis of diabetes, 45 studies (63.38%) were diagnosed by history of past illness, 41 studies (57.75%) were diagnosed by delayed gastric emptying and 30 studies (42.25%) were diagnosed by scores. Moreover, only 13 studies (18.31%) were diagnosed by meeting the diagnostic criteria of related diseases. In addition, only one study (1.41%) did not specify the diagnostic criteria. Of the 71 studies, only the intervention population of 7 studies (9.86%) were diabetic gastrointestinal diseases, and the rest were diabetic gastroparesis. It could be seen that the studies of diabetic gastroparesis occupied predominance. Among them, 31 studies (43.66%) did not mention the severity of diabetic gastroparesis, 14 studies (19.72%) were mentioned severity as moderate to severe and 11 studies (15.49%) were mild to severe. Of the 71 studies, the participants were mostly concentrated in 30 and less (n=24, 33.8%) and 101 to 300 (n=15, 21.13%). Among them, the target sample size of 47 clinical trials (66%) was less than 100. Only 7 studies (9.86%) had more than 300 participants. In addition, of the 71 studies, the majority of the participants were adults and the elderly (n=66, 92.96%), and only 4 studies (5.63%) were adults. Furthermore, 66 studies (92.96%) were unlimited gender.

### 3.3 Intervention characteristics for studies on diabetic gastrointestinal diseases

Intervention characteristics for studies on diabetic gastrointestinal diseases were listed in **Table 2**. Of the 71 included studies, the intervention time of 24 studies (33.8%) was 4 weeks and less and 18 studies (25.35%) reached 12 weeks and above. Among them, only the intervention time of 2 studies (2.82%) was not necessarily. Through statistical analysis, it was found that most studies the common used of short intervention periods. In terms of the follow-up time of the studies, 62 studies (87.32%) were not followed up, and the follow-up times of only studies were concentrated in 14 days and less (n=4, 5.63%) and 4 weeks and above (n=4, 5.63%). Among them, only one study (1.41%) had an irregular follow-up time. There were eight types for the intervention or treatment of patients with diabetic gastrointestinal diseases: Drug; Iiquid Nutrient; Fecal Microbiota Transplantation; Acupuncture; Inspection/Equipment; Diet; Other and no interventions. Of the 71 studies, 44 studies (61.97%) focused on drugs interventions, followed by 15.49% focusing on Inspection/Equipment (n=11), and 8.45% focusing on acupuncture interventions (n=6). As shown in **Figure 2**, the 44 drug intervention trials included metoclopramide (5), prokinetic (19), gastric ghrelin receptor agonists (5), GLP-1 Antagonist (1), motilin receptor agonist (3), gastric accommodation drugs (2), eluxadoline (1), traditional chinese medicine/chinese patent medicines (2), rifaximin (1), other drugs (7). Prokinetic drugs were the most common intervention, accounting for 43.18% of drug-related trials.

### 3.4 Study designs for studies on diabetic gastrointestinal diseases

Study designs for studies on diabetic gastrointestinal diseases were listed in **Table 3**. Of the 71 included studies, most were randomized parallel controlled trials (n = 55, 77.46% VS n = 43, 60.56% VS n = 41, 60.56%). Moreover, 18 studies (25.35%) had a double-blinded research design, while 16 studies (22.54%) did not, and 6 studies failed to provide a description. In addition, 41 studies (57.75%) provided placebo, while 30 studies (42.25%) did not. Of the 71 studies, 42 (59.15%) were multi-center studies, 26 (36.62%) were single-center studies, while 3 studies (4.23%) did not.

**Table 3:** Study designs for studies on diabetic gastrointestinal diseases.

Characteristics	Category	N=71	Percentage of Records (%)
Allocation	Randomized	55	77.46
	Parallel	43	60.56
	Cross-over	11	15.49
	Non-randomised	7	9.86
	Case-Only	1	1.41
	Single Group	9	12.68
	Sequential Assignment	3	4.23
	Case-Control	1	1.41
Masking/Blinding	Single blind	8	11.27
	Double blind	18	25.35
	Triple blind	11	15.49
	Quadruple blind	12	16.90
	Open Label	16	22.54
	N/A	6	8.45
Placebo comparator	Yes	41	57.75
	No	30	42.25
Participating center	Single center	26	36.62
	Multicenter	42	59.15
	N/A	3	4.23

N/A indicates no.

**Table 4:** Outcome measures for studies on diabetic gastrointestinal diseases.

Characteristics	Category	N=71	Percentage of Records (%)
Primary Evaluation indexes	Scores	30	42.25
	Gastric emptying	14	19.72
	Clinical symptoms	7	9.86
	Laboratory examination	5	7.04
	Safety and tolerability	4	5.63
	Number or percentage or proportion of participants	10	14.08
	Time percentage or frequency or proportion	7	9.86
	Gastrointestinal tract	5	7.04
	Others	4	5.63
	N/A	2	2.82
Secondary evaluation indexes	Score	34	47.89
	Laboratory examination	23	32.39
	Gastric emptying	16	22.54
	Safety and tolerability	17	23.94
	Symptoms	16	22.54
	Number or percentage or proportion of participants	11	15.49
	Time percentage or incidence or frequency or proportion	7	9.86
	Pharmacokinetics	5	7.04
	Gastrointestinal tract	6	8.45
	Others	16	22.54
	N/A	13	18.31
Safety indexes	Adverse reactions	3.00	4.23
	Adverse events, Serious adverse events	22.00	30.99
	Safety and tolerability	10.00	14.08
	N/A	44.00	61.97

N/A indicates no.

### 3.5 Outcome measures for studies on diabetic gastrointestinal diseases

Outcome measures for studies on diabetic gastrointestinal diseases were listed in **Table 4**. Of the 71 included studies, the primary and secondary evaluation indexes were based on the scores (n = 30, 42.25% VS n = 34, 47.89%) as the primary index. In terms of primary evaluation indexes, 14 studies (19.72%) were evaluated by gastric emptying, 10 studies (14.08%) were evaluated by the number or percentage or proportion of participants, while 2 studies (2.82%) did not. In terms of secondary evaluation indexes, 23 studies (32.39%) were evaluated by laboratory tests, 17 studies (23.94%) were evaluated by safety and tolerability. In addition, 13 studies (18.31%) failed to provide explanations.

Of the 71 studies, 22 studies (30.99%) were evaluated by adverse events and serious adverse events, 10 studies (14.08%) were evaluated by safety and tolerability. Moreover, 3 studies (4.23%) were evaluated by adverse reactions. The other, 44 studies (61.97%) were not mentioned. Through statistics, it was found that the safety evaluations rate of the studies was low.

## 4 Discussion

The aim of this study was to summarize the clinical research status of diabetic gastrointestinal diseases, in order to provide clinicians with a faster and more convenient understanding of the current research situation. To the best of our knowledge, this is the first comprehensive assessment of the characteristics of diabetic gastrointestinal diseases-related clinical trials. Our results showed that only 13 studies were diagnosed by meeting the diagnostic criteria for related diseases. In the diagnosis of diabetic gastrointestinal diseases, 85.92% of the studies focused on the first diagnosed as diabetes, and then combined with other indications for further diagnosis of diabetic gastrointestinal diseases. After the diagnosis of diabetes, 86 of the studies were based on past medical history, or the participants had delayed gastric emptying diagnosed as diabetic gastrointestinal diseases. In addition, scoring according to the scales or excluding obstruction was also a common method in the diagnosis of diabetic gastrointestinal diseases. Diabetic gastrointestinal diseases are used to describe the gastrointestinal manifestations of diabetes (4). Therefore, diabetic gastrointestinal diseases are a general term, not refer to a single disease. Thus, the diagnostic criteria for diabetic gastrointestinal diseases were slightly different. From the current research, the diagnostic criteria of diabetic gastrointestinal diseases are diverse and more focused on diabetic gastroparesis. The results of this study are similar to the latest clinical research design of diabetic gastroparesis (16), such as diagnostic criteria, safety evaluations, etc. The latest review of diabetic gastroparesis focuses more on pathogenesis and management, while this study focuses more on study designs (13). In terms of diagnostic criteria, this study is similar to the latest review of diabetic gastroparesis (13). However, this study is different from the latest review of enteric neuropathy in diabetes (14). The latest review of diabetic enteric neuropathy focuses more on the specific approaches of diagnosis and pathophysiological mechanisms (14). Furthermore, only 7 studies had studied diabetic gastrointestinal diseases except diabetic gastroparesis. Therefore, diabetic gastrointestinal diseases remain to be further studied, and more studies on diabetic gastrointestinal diseases such as diabetic enteropathy, diabetic dyspepsia and so on can be carried out in the future.

Surprisingly, 66.19% of the studies had a small sample size (≤100). This may be due to many factors can lead to gastrointestinal symptoms (17, 18), which are difficult to distinguish from the gastrointestinal manifestations caused by diabetes. Therefore, the recruitment of participants in the trial was limited. In addition, all participants in the study were satisfied 18 years old. And the age of most participants was spaned two age groups, only 4 studies brought into adults. This may be related to the course of diabetes. Diabetic patients with a long history were prone to diabetic gastroparesis (19). Another potential reason may be that the current studies focused on diabetic gastroparesis. Therefore, the participants were all aged 18 years and above. In addition, the intervention time of 53.52% of the studies focused on 1 month and less, and only 25.35% of the studies lasted for 3 months. This may be related to the compliance of the participants. The cycle of experimental intervention was long, and patient compliance was relatively poor (20, 21), which affected the results and process of the trials, thus limited the intervention time of the trials. Moreover, 62 studies were not followed up, which may be related to the cost of a lot of manpower and time. Meantime, the financial burden on researchers may also limit the follow-up. Therefore, more attention should be paid to the formulation and implementation of diabetic gastrointestinal diseases program.

This study found that 43.18% of the studies focused on prokinetic drugs therapy. Drug-related treatment, especially prokinetic drugs therapy, has been widely used in diabetic gastroparesis (22). Pharmacologic treatment with prokinetics to increase gastric motility formed the mainstay for the treatment of diabetic gastroparesis (23). However, the use of prokinetics was limited by adverse effects and serious adverse effects (23). Therefore, more studies are needed in the future to carry out the effectiveness of other interventions for diabetic gastroparesis or diabetic gastrointestinal diseases in clinical practice.

Many studies included strong design elements such as randomization, parallel, double blinding, and placebo control groups. In addition, 15.49% of the studies implemented cross-over design. This study design can better measure the effect of the trials and improve patients compliance. Furthermore, 69.01% of the studies used different methods of blinding. The experimental design of the blind method could reduce the difference assessment of outcomes and avoid analytic bias (24). Surprisingly, the number of double-blind and open study designs was almost the same, accounting for 47.89% of the entire studies. The primary and secondary evaluation indicators of the studies contained similar content, but the focus was slightly different. This may be related to the different purposes of the studies. In addition, there were no secondary evaluation indicators in 13 studies. It is recommended to increase the evaluation of secondary relevant indicators to expand the richness and depth of studies. Moreover, the secondary evaluation indicators of 22.54% of the studies could not be classified because there were no similarities. Meantime, 90.15% of the studies were diabetic gastroparesis. It can be seen that there is no consensus on the evaluation indexes of diabetic gastroparesis at present. It is recommended to further explore its related evaluation indicators in the future to form a relatively standardized and comprehensive unified efficacy evaluation. Surprisingly, 61.97% of the studies did not conduct safety evaluation. This may be related to factors such as funding limitations or study design constraints. The safety of the intervention remained to be further investigated, and it was also important to continue to report the safety of the trials. To observe the occurrence of adverse events in the study participants, whether slight or serious, safety evaluation is an important part of the efficacy evaluation indexes. Therefore, it is suggested that future studies should take safety evaluation as a part of efficacy evaluations. However, small sample sizes, short intervention durations and inconsistent safety evaluations may influence the generalizability of findings, thereby hindering their translation into clinical applications. Moreover, there are also considerable heterogeneity in diagnostic criteria and intervention methods, which will have a certain influence on the treatment, management and development of guidelines for patients with diabetic gastrointestinal diseases in the future.

This summary for diabetic gastrointestinal diseases clinical trials had several limitations. Firstly, there may be potential publication bias, such as registry data incompleteness, and the exclusion of unpublished or non-English studies. Secondly, although as widely as possible to retrieve the relevant studies, there may still be missed trials, which may lead to selection bias and reporting bias. These factors might lead to deviations and non-universality in the results.

## 5 Conclusion

This study presented the first comprehensive overview of the clinical research status of diabetic gastrointestinal diseases. In addition, this study played a key role in pinpointing methodological shortcomings in diabetic gastrointestinal diseases trials. It also paved the way for more robust future research. Our results indicated that the diagnostic criteria of diabetic gastrointestinal diseases are diverse and more focused on diabetic gastroparesis. In the future, researchers should carry out more studies on diabetic gastrointestinal diseases other than diabetic gastroparesis. In addition, most studies had small sample size, short intervention time, large differences in secondary evaluation indicators, and less follow-up. It is recommended to the development of standardized diagnostic protocols and perform longer follow-up periods. Meantime, it is also recommended that comprehensive safety endpoints be included in future trial designs to provide high-quality clinical evidence. Furthermore, the longer the duration of diabetes, the more easy to develop related complications. Therefore, it is not only necessary to pay more attention to patients with diabetic gastrointestinal diseases, but also to pay more attention to diabetic patients in daily care. By conducting large-sample, long-term, multi-angle clinical studies, patient outcomes and the development of evidence-based guidelines can be further improved. Meanwhile, enhanced standardization of study methodology and comprehensiveness of result evaluation are clearly warranted for continued improvements to studies in this field. In addition, the efficacy of different methods in the treatment of diabetic gastrointestinal diseases can be further explored through systematic evaluation and or meta-analysis in the future.

## Data Availability

The original contributions presented in the study are included in the article/**Supplementary Material**. Further inquiries can be directed to the corresponding authors.
